# Admission hyperdense sinus sign predicts poorer outcomes in cerebral venous sinus thrombosis

**DOI:** 10.1007/s00415-026-13660-0

**Published:** 2026-02-07

**Authors:** Asaf Honig, Ruth Eliahou, Naaem Simaan, Hen Hallevi, Issa Metanis, Rom Mendel, Rani Barnea, Eitan Auriel, Jonathan Naftali, Shorooq Aladdin, David Orion, Ronen R. Leker, Jeremy Molad

**Affiliations:** 1 The Faculty of Health Sciences, Ben-Gurion University of the Negev, Be’er-Sheva, Israel; 2 Department of Neurology, Soroka University Medical Center, Be’er-Sheva, Israel; 3 Departments of Radiology, Rabin Medical Center, Petach-Tikva, Israel; 4 Department of Neurology, Ziv Medical Center, Safed, Israel; 5 The Azrieli Faculty of Medicine, Bar Ilan University’s, Safed, Israel; 6 Department of Neurology & Stroke, Tel-Aviv Sourasky Medical Center, Tel-Aviv, Israel; 7 Gray Faculty of Medicine, Tel-Aviv University, Tel-Aviv, Israel; 8 Department of Neurology, Hadassah-Hebrew University Medical Center, Jerusalem, Israel; 9 Department of Neurology, Assuta Medical Center, Ashdod, Israel; 10 Departments of Neurology, Rabin Medical Center, Petach-Tikva, Israel; 11 Departments of Neurology, Sheba Medical Center, Ramat-Gan, Israel

**Keywords:** Cerebral sinus venous thrombosis, Hyperdense sinus sign, Dense vessel sign, Acute symptomatic seizure, Papilledema

## Abstract

**Background:**

The hyperdense sinus sign (HDSS) is a readily identifiable non-contrast CT marker of acute thrombus in cerebral venous sinus thrombosis (CVST). We aimed to characterize HDSS associated features and prognostic significance.

**Methods:**

Data from prospective multicenter CVST registries was analysed. HDSS was defined as attenuation > 70 Hounsfield units within a thrombosed venous structure. Baseline characteristics and outcomes were compared between patients with and without HDSS on admission CT. Multivariable logistic regression identified independent predictors of Excellent-Functional-Outcome (mRS 0–1) and remote seizures.

**Results:**

Among 465 patients (mean age 41.9 ± 18.4 years; 64.3% female), 178 (38.3%) exhibited HDSS. Patients with HDSS had higher rates of oral contraceptives use (28% vs 18%, p = 0.009), seizures at presentation (23% vs 14%, p = 0.015), superior sagittal (45% vs 35%, p = 0.028) and transverse sinus involvement (78% vs 67%, p = 0.01), deep venous thrombosis (8% vs 2%, p = 0.003), cortical vein thrombosis (19% vs 9%, p = 0.004), and multisite occlusion (34% vs 22%, p = 0.002). Parenchymal lesions were more common in HDSS patients, including intracerebral hemorrhage (27% vs 13%, p < 0.001) and venous infarction (22% vs 11%, p = 0.004). On day-90, HDSS was associated with lower Excellent-Functional-Outcome rates (71% vs 82%, p = 0.022), higher rates of remote seizures (9% vs 3%, p = 0.001), and similar recanalization rates. HDSS independently predicted reduced odds of Excellent-Functional-Outcome (OR = 0.491 [0.261–0.926], p = 0.028) and increased remote seizures (OR = 2.693 [1.057–6.861], p = 0.038).

**Conclusions:**

HDSS identifies a CVST subgroup with more extensive thrombosis, greater parenchymal injury, and poorer outcomes, supporting its utility in early risk stratification.

**Supplementary Information:**

The online version contains supplementary material available at 10.1007/s00415-026-13660-0.

## Introduction

Cerebral venous sinus thrombosis (CVST) is an uncommon but clinically heterogeneous cause of stroke. Although timely diagnosis and anticoagulation remain the cornerstone of management, the clinical course is variable, and early identification of patients at risk for an unfavorable outcome is a major challenge. Noncontrast CT (NCCT), routinely performed as the first-line neuroimaging study in suspected CVST, provides not only diagnostic but also potential prognostic information that is frequently underrecognized.

The **hyperdense sinus sign (HDSS)** is a simple NCCT finding representing intraluminal thrombus within the dural venous sinuses, analogous to the hyperdense vessel sign in acute arterial stroke [1–4]. This sign reflects acute clot formation with high attenuation due to red blood cell–rich content or elevated hematocrit and typically resolves within days to weeks as the thrombus organizes or recanalizes [5]. When observed in the superior sagittal sinus, it has been referred to as the * “cord sign” [3, 4]. Although HDSS is easily detectable in routine practice, its clinical implications remain uncertain.

Physiologic or systemic factors such as polycythemia, dehydration, or generalized high hematocrit may produce diffuse venous hyperattenuation and should be differentiated from true HDSS [5]. Beyond its diagnostic value, however, the presence of HDSS may identify patients with recent, extensive, or more aggressive thrombotic disease, potentially predisposing to parenchymal injury and adverse outcomes.

Given the limited evidence regarding its prognostic meaning, we aimed to systematically evaluate the clinical and radiologic characteristics associated with HDSS and determine its relationship with functional outcome, recanalization, and seizure occurrence in a large multicenter cohort of patients with CVST.

## Methods

### The dataset

A retrospective analysis was performed on a dataset including patients diagnosed with CSVT from six comprehensive stroke centers between 1/2010 and 12/2023. The dataset was collected in an ongoing prospective manner. We included in this analysis patients diagnosed with CVST aged 18 and above, excluding cases in which CSVT was secondary to head trauma. Data regarding patient demographics, possible etiologies, comorbidities, and vascular risk factors was recorded. We have used the STROBE [6] guidelines for multicenter cohort observational studies while preparing the manuscript.

### CSVT diagnosis

The diagnosis of CSVT in all included cases was based on cerebrovascular imaging (CT venography, MR venography, or digital subtraction angiography).

### HDSS definition

**Fig. 1:**
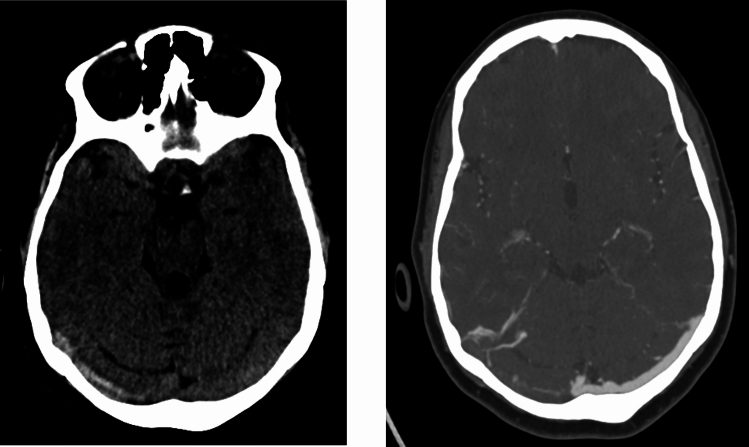
Hyperdense Sinus Sign. Non-contrast CT demonstrates hyperdense sinus sign in the right transverse sinus (left panel) and corresponding thrombosis on CT-venography (right panel)

A prior study evaluated two attenuation thresholds on non-contrast CT for the diagnosis of CSVT [7]. A cutoff of > 70 Hounsfield units (HU) reliably indicated venous thrombosis but failed to detect approximately 20% of thrombosed sinuses. In contrast, in that study a threshold ≥ 60 HU increased sensitivity but produced several false positives in patients without venous sinus thrombosis. Given that our objective was to assess the role of HDSS in patients with an already established diagnosis of CSVT, we adopted the higher threshold. Accordingly, HDSS was defined as venous sinus hyperdensity with an attenuation > 70 HU on admission NCCT (Fig. 1). HU values were measured at the site of highest density on NCCT on three points of each thrombosed sinus. Thrombosis was confirmed through subsequent venous imaging and comparison of attenuation values with normal-appearing sinuses. Evaluated were superior sagittal sinus, transverse sinus, sigmoid sinus, distal jugular vein and deep cerebral veins. Potential mimics, including vascular calcification, elevated hematocrit, and intravenous contrast, were excluded at all participating centers by a certified neuroradiologist and the site primary investigator. An HDSS-positive CVST patient was defined as one exhibiting hyperdensity in at least one venous sinus. To reduce potential bias arising from heterogeneity in imaging acquisition protocols, we restricted the HDSS analysis to patients imaged during recent years.

Among the 566 CSVT patients in our consortium database, the most recent (January 2014 onwards) 465 consecutive cases were reviewed for the presence of HDSS and included in this study. No significant differences were observed between the 101 patients not reviewed for HDSS and the 465 patients with available HDSS status regarding baseline characteristics, involved sinuses, or prognostic features.

To assess the reliability of HDSS diagnosis, baseline non-contrast CT scans from two major centers (n = 271, 58.3%) underwent central review in a core laboratory by an experienced neuroradiologist blinded to both clinical data and CTV findings. Inter-rater agreement yielded a Cohen’s kappa coefficient of 0.812 (SE = 0.068, p < 0.001), indicating excellent reliability.

### Additional medical data

In all patients, a complete blood count, routine blood biochemistry, and coagulation profiles were performed. The supplementary materials include details of additional radiological, hematological, rheumatological, and oncological investigations.

### Follow-up data

Follow-up neuroimaging of either CTV or MRV was performed three months post admission. Similar to previous definitions [8], degree of recanalization, when specified, was classified as none, partial, or complete. Any recanalization was regarded as the combination of both partial and complete recanalization. Patient follow-up included a 3 post admission visit at the stroke prevention clinics of the participating centers. Functional independence on follow-up was graded using the modified Rankin Score (mRS) [9]. Excellent outcome was defined as mRS 0–1 on 90-day follow-up. Mortality was documented after 12 months. Remote seizures were defined as any seizure beyond the first week from admission [10]. Recurrent CSVT was defined as the presence of new thrombus in a remote site of the cerebral venous sinuses or cortical veins at least 1 month after admission, or as worsening or extension of a previously diagnosed thrombus despite anticoagulation therapy after prior radiological evidence of complete or partial recanalization.

### Statistical analysis

In the current analysis, we compared data for CVST patients with and without HDSS on admission noncontrast CT of head. Statistical analysis was performed with SPSS version 25 (IBM, Chicago, IL, USA). The two-sample Student's T-test was applied to test differences between the study groups for quantitative parameters. Nonparametric tests were used for the comparison of medians with interquartile ratios. Pearson’s chi-square or Fisher’s exact tests were applied for testing the differences between the groups for the categorical parameters. A p value ≤ 0.05 was considered statistically significant. Multivariate backwards elimination regression models controlling for age, sex, venous infarction, and all variables that yielded a p value < 0.05 were used to control possible confounders.

### Ethical approval

Ethical approval was obtained from the institutional review boards of all participating centers, which granted a waiver of individual informed consent due to the use of anonymized data and the retrospective design of the study.

## Results

465 CSVT patients were included in this study (mean age: 41.9 ± 18.37 years; 64.3% female). Complete follow-up information including mRS-90 was available for 449 patients (96.6%). Compared with patients without any HDSS, those with at least one venous structure qualifying for HDSS (n = 178, 38.3%), had higher rates of oral contraceptives administration (28% versus 18%, p = 0.009) and lower rates of factor V Leiden deficiency (4% versus 11%, p = 0.009) but all other baseline characteristics and underlying CSVT etiology were similar (Table 1). Patients with HDSS had higher rates of seizures at presentation (23% versus 14%, p = 0.015), higher rates of come (9% versus 2.3%, p = 0.007), and lower rates of headache (71% versus 81%, p = 0.006). HDSS was associated with higher rates of SSS (45% versus 35%, p = 0.028), transverse sinus (78% versus 67%, p = 0.01), deep venous involvement (8% versus 2%, p = 0.003), cortical vein thrombosis (19% versus 9%, p = 0.004) as well multi-site involvement (34% versus 22%, p = 0.002). Among patients with multi-site involvement, 77% had HDSS in more than one site. HDSS was also associated with higher rates of ICH (27% versus 13%, p < 0.001) and venous infarction (22% versus 11%, p = 0.004).

**Table 1:** Baseline characteristics of CVST patients hyperdense sinus sign

Characteristics	W/O hyperdense sinus sign N = 287	Hyperdense sinus sign N = 178	P value
Age, mean (SD)	42.2 (18.46)	41.42 (18.28)	0.441
Gender, male (%)	103 (36)	63 (35)	0.914
Dehydration (%)	9 (3)	6 (3)	0.889
Neurosurgery (%)	7 (2)	1 (1)	0.206
Epidural (%)	10 (3)	2 (1)	0.119
Infections (%)	20 (7)	10 (6)	0.564
Smoking (%)	57 (20)	35 (20)	0.952
Hyperlipidemia (%)	37 (13)	25 (14)	0.722
Hypertension (%)	35 (12)	26 (15)	0.463
Obesity* (%)	8 (2)	8 (4)	0.142
Diabetes (%)	21 (7)	16 (9)	0.524
Active malignancy (%)	44 (15)	22 (12)	0.372
Previous thrombotic events (%)	28 (10)	16 (9)	0.789
Behcet's disease (%)	13 (5)	4 (2)	0.208
Inflammatory bowel disease (%)	6 (2)	4 (2)	0.655
Spontaneous abortions (%)	5 (2)	4 (2)	0.443
Pregnancy/post-partum	24 (8)	11 (6)	0.386
IVF	5 (2)	5 (3)	0.441
Oral contraceptives (%)	51 (18)	49 (28)	**0.009**
Clinical presentation			
Papilledema (%)	81 (28)	38 (21)	0.102
Headache (%)	232 (81)	126 (71)	**0.006**
Vomiting (%)	66 (23)	33 (19)	0.946
Any focal neurological deficit (%)	72 (25)	61 (34)	0.083
Seizure at presentation (%)	41 (14)	41 (23)	**0.015**
NIHSS admission, (IQR)	0 (0–0)	0 (0–1)	**0.015**
Coma (%)	16 (9)	33 (19)	**0.007**
Hematological workup			
APLA (%)	32 (11)	17 (10)	0.585
Protein C/S deficiency (%)	4 (1)	16 (9)	0.051
Factor V deficiency (%)	31 (11)	7 (4)	**0.009**
Factor II mutation (%)	4 (1)	3 (2)	0.799
PT 20210	11 (4)	12 (7)	0.143
MTHFR (%)	16 (6)	4 (2)	0.084
JAK 2 (%)	21 (7)	13 (7)	0.587
Thrombocytosis (%)	21 (7)	9 (5)	0.32
Hyperhomocisteinemia (%)	7 (2)	2 (1)	0.326
Radiological findings			
Multiple veins (%)	62 (22)	61 (34)	**0.002**
Venous infarction (%)	33 (11)	39 (22)	**0.004**
ICH (%)	36 (13)	48 (27)	** < 0.001**
Any recanalization (%)	192 (67)	119 (67)	0.773
Complete recanalization (%)	111 (39)	63 (35)	0.523
Involved sinus			
Superior sagittal sinus (%)	100 (35)	80 (45)	**0.028**
Transverse sinus (%)	191 (67)	138 (78)	**0.01**
Sigmoid sinus (%)	188 (66)	119 (67)	0.74
Cavernous sinus (%)	6 (2)	3 (2)	0.753
Cortical (%)	27 (9)	33 (19)	**0.004**
Deep (%)	7 (2)	15 (8)	**0.003**
Prognosis			
MRS 90 (IQR)	0 (0–1)	0 (0–1)	**0.015**
Excellent outcome (%)	234 (82)	127 (71)	**0.022**
Mortality (%)	15 (5)	10 (6)	0.823
Remote seizures (%)	10 (3)	16 (9)	**0.001**
Recurrent CSVT (%)	8 (3)	6 (3)	0.704

Obesity was defined as body mass index > 30

P-values that are statistically significant are highlighted in bold

CSVT cerebral sinus venous thrombosis, *APLA* antiphospholipid syndrome

HDSS was associated with worse outcomes, including lower rates of an excellent outcome (71% versus 82%, p = 0.022), worse mRS-90 (p = 0.015) and higher rates of remote seizures (9% versus 3%, p = 0.001). No differences were found in the rates of either complete or partial recanalization on 90-day follow-up imaging. A multivariate analysis was conducted, adjusting for all variables significantly associated with prognostic outcomes in univariate analysis. Our model accounted for age, sex, oral contraceptive use, Factor V Leiden deficiency, seizure at presentation, HDSS presence, thrombosis involving the SSS, transverse sinus, or deep CSVT, multiple vein involvement, and venous infarct or ICH (Table 2). HDSS remained significantly associated with lower rates of excellent outcome (OR = 0.491 [0.261–0.926], p = 0.028).

**Table 2:** Multivariate analysis – predictors for excellent outcome (mRS 0–1 on day 90)

	OR	95% Confidence interval	p value
Age	0.946	0.930–0.962	** < 0.001**
Sex (male)	1.524	0.797–2.915	0.202
Seizure at presentation	0.243	0.113–0.509	** < 0.001**
Superior sagittal sinus involvement	0.522	0.234–1.167	0.113
Transverse sinus involvement	1.172	0.449–3.012	0.704
Deep CSVT	0.233	0.067–0.811	**0.022**
Multiple vein involvement	1.164	0.449–3.012	0.755
HDSS	0.491	0.261–0.956	**0.028**
Venous infract	0.698	0.474–1.029	0.07

P-values that are statistically significant are highlighted in bold

CSVT cerebral sinus venous thrombosis, *HDSS *hyperdense sinus sign

In the multivariable analysis examining predictors of remote seizures, the presence of the hyperdense sinus sign (HDSS) remained independently associated with subsequent seizure occurrence (OR = 2.693 [1.057–6.861], p = 0.038) (Table 3). Moreover, the only additional independent predictor of remote seizures was seizure at presentation (OR = 12.186 [4.552–32.169], p < 0.001).

**Table 3:** Multivariate analysis for predictors of remote seizures (beyond first week of diagnosis)

Variable	OR	95% Confidence interval	p value
Male sex	1.988	0.793–4.985	0.143
Age	0.99	0.965–1.016	0.445
Seizure at presentation	12.186	4.552–32.169	** <.001**
Venous infarct	1.10	0.518–2.333	0.804
Intracerebral hemorrhage	.618	0.199–1.924	0.406
Cortical	1.399	0.484–4.045	0.536
HDSS	2.693	1.057–6.861	**0.038**

P-values that are statistically significant are highlighted in bold

CSVT cerebral sinus venous thrombosis, *HDSS *hyperdense sinus sign

We analyzed the proportion of thrombosed venous structures meeting the criteria for HDSS (Table 4). Approximately half of the thrombosed superior sagittal sinuses (SSS) qualified for HDSS, compared with only about one quarter of thrombosed sigmoid sinuses (*p* < 0.01). In addition, the mean Hounsfield unit (HU) values were significantly higher in thrombosed SSS segments than in the sigmoid, jugular, and deep venous structures (*p* < 0.01 for all), and showed a non-significant trend toward higher values compared with the transverse sinus (*p* = 0.06) (Table 4).

**Table 4:** Hyperdensity characteristics of thrombosed cerebral venous sinuses

Cerebral venous structure	Thrombosed sinuses meeting HDSS definition (%)	Mean ± SD HU measurement
Superior sagittal sinus	50	70.4 ± 12.1
Transverse sinus	40	65.6 ± 12.3
Jugular vein	34.8	62.7 ± 14.4
Sigmoid sinus	25.8	58.7 ± 15.6
Deep veins	20	66 ± 6.3

*HDSS *hyperdense sinus sign, *HU *hounsfield units

## Discussion

Our study found that the presence of admission HDSS was associated with a more fulminant CVST clinical presentation. This is explained by more profound radiological findings such as higher rates of ICH, venous infarction and multiple vein involvement which are all well documented predictors of poor outcomes. [11–13] Moreover, the parenchymal involvement has resulted in an independent association of HDSS with remote seizures [14]. Several mechanisms may explain this association. We propose that HDSS serves as a marker of recent thrombosis, with its presence alongside multiple vein involvement suggesting a rapid progression of thrombus formation. In such cases, compensatory mechanisms such as increased collateral venous drainage may fail to adapt, leading to parenchymal injury. A more gradual course of illness may allow the venous collaterals can adapt by dilating veins and capillaries, reversing distal venous flow, and recruiting adjacent venous territories to mitigate the effects of venous obstruction [15]. The variability in venous collateral status among individuals may explain the differing outcomes observed in CVT patients. In some cases, the venous collateral circulation is sufficient to overcome the obstruction without causing significant clinical symptoms, while in others, inadequate collateralization may result in permanent parenchymal injury [16]. This hypothesis aligns with a study examining the time from symptom onset to admission, which found that patients presenting earlier were more likely to experience seizures, focal neurological deficits, and exhibit parenchymal lesions on initial imaging [17].

Another possible mechanism is the presumed relatively rapid clot extension associated with HDSS. Such extension may involve either cortical or deep sinuses resulting in worse prognostic outcomes. Previous studies showed that cortical vein involvement, due to its location on the cerebral cortex is an independent predictor of seizures and parenchymal damage [18]. Similarly, deep CSVT has been independently associated with worse functional outcomes [19].

Surprisingly, despite the assumption that HDSS represents a more recent clot, it did not correlate with higher rates of complete or partial recanalization. One possible explanation is that HDSS patients had more extensive clot burden with more thrombosed sinuses on presentation, thus failing to achieve clot fragmentation and goals of sinus recanalization [8]. While a recently published study looked at early recanalization, [20] our study lacks this data. Alternatively, HDSS may have an underlying etiology rendering them less amenable to early recanalization.

Patients with HDSS had higher rates of exogenous estrogen-containing oral contraceptive use. Estrogen increases the risk of cerebral sinus venous thrombosis (CSVT) through several mechanisms. Its procoagulant effects include elevated levels of clotting factors such as fibrinogen, factor VII, factor VIII, and factor X, along with reduced levels of anticoagulant factors like protein S [15]. Additionally, estrogen is associated with reduced fibrinolysis by increasing plasminogen activator inhibitor-1 (PAI-1), which inhibits clot breakdown [15]. Exogenous estrogen may also promote endothelial dysfunction by activating endothelial cells and enhancing the expression of adhesion molecules [16]. Finally, it may interfere with the physiological autoregulation of cerebral vasculature [17].

In previous studies of the hyperdense artery sign (HDAS), clot histology demonstrated a higher red blood cell (RBC) content with less fibrin. These RBC-rich clots were more easily retrieved using aspiration alone, without the need for a stent retriever, possibly indicating reduced adhesion to the vessel wall. A large clot histology-based meta-analysis found that RBC-rich thrombi were associated with higher recanalization rates and shorter procedure times [1]. However, our findings showed that patients with HDAS had comparable rates of partial and complete recanalization, suggesting the involvement of other pathophysiological mechanisms. Therefore, tailored strategies to enhance clot degradation should be considered in patients with HDSS.

Taken together, our findings indicate that HDSS was most frequently observed and most pronounced in the superior sagittal sinus, corresponding to higher clot density and more extensive thrombosis. Although we cannot determine the temporal stage of thrombosis, these site-specific density differences may suggest variation in thrombus composition or flow dynamics across venous territories. Recognizing such anatomical and radiological distinctions could improve early imaging interpretation and contribute to future efforts toward more refined prognostic stratification in CVST.

The strengths of our study include the relatively large multi-center consecutively enrolled cohort of CSVT patients, the vast clinical and radiological features described, and the high rate of clinical follow-up. However, our study has several limitations. First, the retrospective design of the study is prone to different types of bias. Second, 101 patients were excluded from the current analysis as the presence of HDSS was not evaluated. However, as described, no significant differences were found between included and excluded patients. Third, we had no documentation regarding symptoms onset time before admission, which may be in association with HDSS. Fourth, Routine follow-up was performed 3 months post-admission. The study could have benefit from longer follow-up period, as it is reasonable that some of the patients will further improve. Last, MRI was not routinely performed during admission, and therefore we cannot rule out the possibility of co-pathologies.

## Supplementary Information

Below is the link to the electronic supplementary material.

Supplementary file1 (DOCX 15 KB)

## Data Availability

The data that support the findings of this study are available from the corresponding author upon reasonable request. Full data is available following a formal reasonable request and in compliance with state and participating centers regulations**.**
