# Supplementary material for: Admission hyperdense sinus sign predicts poorer outcomes in cerebral venous sinus thrombosis
Source: J Neurol. 2026 Feb 7;273(2):120. doi: 10.1007/s00415-026-13660-0 (PMC12882849; doi:10.1007/s00415-026-13660-0)
Supplement: Supplementary file 1 — Supplementary file1 (DOCX 15 KB) [file 415_2026_13660_MOESM1_ESM.docx]

Supplemental materials:

The following investigations were conducted:

Radiological assessment: These were interpreted by both an experienced neuroradiologist and an experienced stroke neurologist. The side and location of the occluding venous channels were reported and scored as following: 1. Superior sagittal sinus 2 transverse and sigmoid sinuses either with or without jugular. 3. cavernous sinus 4. deep venous system. The deep venous system was defined to include the internal cerebral veins (veins of Galen and Rosenthal). 5. cortical vein. Multiple vein/sinus involvement was defined as involvement of more than one site according to the above vein scores. The presence of ICH was determined from admission NCCT.

Hematological Workup and Hypercoagulability Evaluation: Routine laboratory screening for hypercoagulable states included tests for protein C, protein S, antithrombin III, factor V Leiden, homocysteine, and antibodies to anticardiolipin, beta-2 glycoprotein 1, and Lupus anti-coagulant (LAC) were taken upon CSVT diagnosis. Laboratory criteria for antiphospholipid syndrome were defined

by at least two positive tests performed at least 12 weeks apart. Tests included LAC RVVT ratio >1.2, LAC SCT >1.23, IgG/IgM anticardiolipin antibodies, and/or IgG/IgM anti–β2-glycoprotein I antibodies ELISA value above 40 units. In patients with thrombocytosis of unknown cause (platelet count >450,000/µL) or unexplained polycythemia (hemoglobin >18 g/dL for males and >16 g/dL for females), genetic analysis for JAK2-V617F mutation was performed.

Rheumatological Assessment: A rheumatologic questionnaire was completed for all patients, documenting any history of arthritis, arthralgia, rash, oral or genital ulcers, and uveitis. Further rheumatologic investigations, including a full rheumatological laboratory workup, were performed when clinically indicated. Pathergy testing and genetic screening for HLA-B27 and HLA-B51 were conducted when Behçet’s disease was suspected.

Neoplastic Investigation: In patients with CVST of unknown etiology, a panel of neoplastic markers was measured, including prostate-specific antigen (PSA), cancer antigen 125 (CA 125), carcinoembryonic antigen (CEA), carbohydrate antigen 19-9 (CA19-9), alpha-fetoprotein (AFP), and human chorionic gonadotropin (HCG). For patients aged ≥50 years, chest, abdomen, and pelvis CT scans were also performed.
